# Does subclinical atrial fibrillation independently predict adverse outcomes in patients with heart failure and cardiac resynchronization therapy defibrillator

**DOI:** 10.1002/clc.23547

**Published:** 2021-01-19

**Authors:** Jie Chen, Peng Yu, Jianyong Ma, Chunhua Zheng, Xiao Liu

**Affiliations:** ^1^ Cardiology Department The Third Affiliated Hospital of Nanchang University Nanchang Jiangxi China; ^2^ Endocrine Department The Second Affiliated Hospital of Nanchang University Nanchang Jiangxi China; ^3^ Department of Pharmacology and Systems Physiology University of Cincinnati College of Medicine Cincinnati Ohio USA; ^4^ Department of Cardiology Sun Yat‐sen Memorial Hospital Guangzhou China

To the Editor:

We read with interest the study by Arai et al^1^ assessing the effects of s device‐detected subclinical atrial fibrillation (AF) on the clinical outcomes in patients with cardiac resynchronization therapy defibrillator.

However, we had several concerns. First, Cox regression was used to estimate the risk ratio of sub‐AF for heart failure (HF) hospitalization in this study. Notably, the application of Cox model in these results might not appropriate. As shown in figure 1, the three curves of these groups were obviously intersected,^1^ which means that the three curves do not meet a proportional hazard assumption. The proportional hazard hypothesis is that the effect of covariates on survival rate does not change over time. However, in the process of survival analysis, the influence of some clinical factors on hazard function will change correspondingly over time; thus, it is necessary to test that the explanatory variables analyzed to satisfy the proportional hazard assumption in the analysis of survival data using the Cox model.^2^ Therefore, we suggested the authors used a time‐dependent Cox regression model to compared the differences between groups in this study.

Second, history of HF independently predicts the worse outcomes in patients with HF. Moreover, a recent article also reported that in patients with a pacemaker or defibrillator, a history of HF was the strongest independent risk factor for HF hospitalization.^3^ On the other hand, it is well known that AF was associated with adverse events, including hospitalization, cardiovascular death, and all‐cause death.^4^ However, as shown in table 1, the history of HF was not presented across the AF status.^1^ Was there a significant difference in the history of HF between the three groups (subclinical AF, clinical AF, no‐AF)? And it was also questionable whether the subclinical AF predicted the HF hospitalization independently of the history of HF. This issue above might be further discussed in their article.

## CONFLICT OF INTEREST

The authors have declared no competing interest.

## Data Availability

Data sharing is not applicable to this article as no new data were created or analyzed in this study.
